# High throughput molecular dynamics for drug discovery

**DOI:** 10.1186/s40203-015-0007-0

**Published:** 2015-02-13

**Authors:** Nathaniel Stanley, Gianni De Fabritiis

**Affiliations:** Computational Biophysics Laboratory (GRIB-IMIM), Universitat Pompeu Fabra, Barcelona Biomedical Research Park (PRBB), C/Doctor Aiguader 88, Barcelona, 08003 Spain; Institució Catalana de Recerca i Estudis Avançats, Passeig Lluis Companys 23, Barcelona, 08010 Spain

**Keywords:** High-throughput molecular dynamics, Markov state models, GPU, Drug discovery, Fragments

## Abstract

Molecular dynamics simulations hold the promise to be an important tool for biological research and drug discovery. Historically, however, there were several obstacles for it to become a practical research tool. Limitations in computer hardware had previously made it difficult to simulate for long enough to see interesting biological processes. Recent improvements in hardware and algorithms have largely removed this issue, leaving data analysis as the main obstacle. Advances in Markov state modeling appear to be on the way to remove this obstacle. We outline these advances here and discuss numerous recent studies that demonstrate that molecular dynamics simulations will start to be an important tool for pharmaceutical research.

## Review

Drug discovery is an iterative process that relies on various computational tools to help both lead experiments and understand data. High throughput virtual screening, docking, and hit development based on structure-activity relationships, are among many tools routinely used to identify or improve potential drug compounds (Jorgensen 2004; Sliwoski et al 2014). Still, most such methods rely on simplified assumptions that come with fundamental limitations. While such simplifications are needed at early stages, as development progresses and the chemical space begins to be narrowed down, methods with more accuracy should be employed (Rastelli et al 2009; Harvey et al 2009).

Molecular dynamics (MD) simulation is one such method. MD simulations combine Newtonian physics and an all-atom, flexible representation of proteins, water and other molecules to understand the dynamic interactions between them. They can provide important qualitative information, such as where and how a drug binds, but also quantitative information like the binding affinities and kinetics of such interactions (Buch et al 2011). This atomic-level description, combined with the ability to compare it quantitatively with experiments has long made MD a very promising method.

Several substantial obstacles have traditionally prevented this from becoming a reality. Even the most basic biological events like side chain flipping or loop motions in a protein take hundreds of nanoseconds or longer (Zwier and Chong 2010). Therefore, many orders of magnitude in simulated time must be resolved in order to see even simple events in a single simulation. It is computationally costly to span so much time, and this has traditionally been what has kept MD from being practically useful for biological research and drug discovery. Enhanced sampling techniques were developed to speed up MD, but they require biasing along a reaction coordinate or prior knowledge of the system, which is many times unknown.

**Figure 1:**
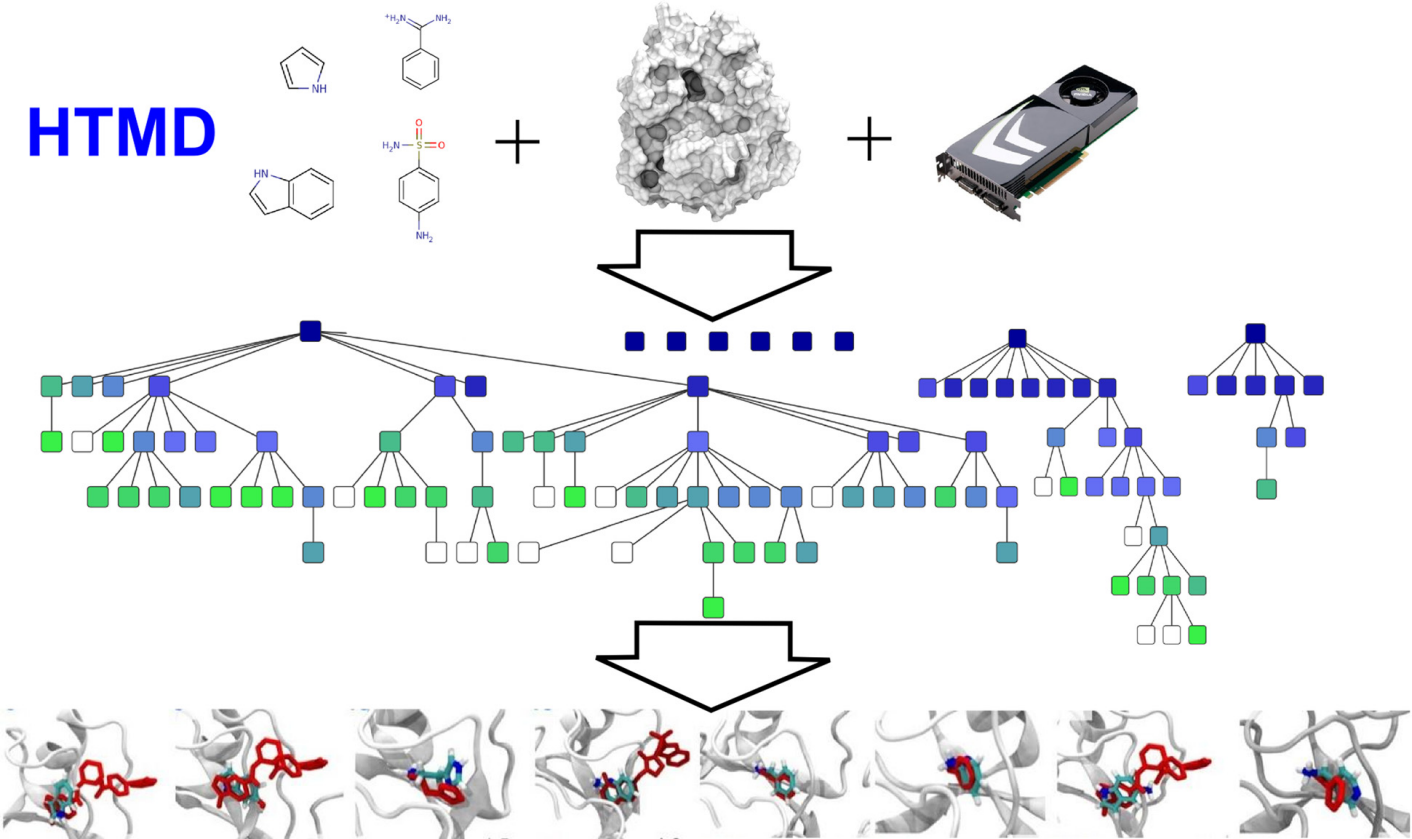
**A high-throughput molecular dynamics workflow.** A protein of interest is selected for study along with potential ligands (if any), and are simulated across multiple parallel runs using GPU devices (top). Additional rounds of simulation are performed, and new simulations may be respawned manually or automatically from previous runs to enhance sampling (middle). In the case of a fragment screen, for example, the result is a series of binding poses which can be compared and contrasted with other methods or used as a basis for lead development (bottom). Affinity and kinetic data are available for each interaction thanks to Markov state modelling.

Further specialized supercomputers, such as the Anton supercomputer (Shaw et al 2007; Shaw et al 2014) or the MD-GRAPE (Ohmura et al 2014), have been developed that can run single simulations on very long timescales, up to a millisecond. A more practical way to approach this problem is to take advantage of recent hardware advances in GPU devices (Harvey et al 2009; Harvey and De Fabritiis 2012). A single GPU can now produce a microsecond simulation in a few days for a small system (~25,000 atoms). Running multiple parallel simulations on a small cluster of GPUs, one can reach millisecond aggregate sampling, a timescale needed to adequately sample many biological processes, including binding of many small molecules (Buch et al 2010). We call this approach high-throughput molecular dynamics, or HTMD (Harvey and De Fabritiis 2012) (Figure 1). Accessibility to such hardware has never been easier to obtain thanks to commodity cloud services like Amazon AWS, so the barrier to entry to employing MD as a standard tool in the drug discovery process has been drastically lowered.

All this increased computational power results in a large amount of data, at which point analysis becomes a major concern. The copious and disjointed nature of the data produced by HTMD studies means that making sense of it is a significant challenge. Clustering methods to understand the data have trouble properly assigning weight and relevance to the data. Further, it is often not clear to newcomers that running multiple parallel simulations can allow one to investigate events that are much slower than the length of each individual simulation. This is indeed possible thanks to Markov state models (MSM), which allow one to take advantage of the statistical probability of events. The basics of MSMs have been covered at length, and we direct the reader to several publications for a more detailed look (Noé and Fischer 2008; Pande et al 2010; Prinz et al 2011). For the remainder of this review, we focus mainly on our experience with these tools and proof-of-concept studies employing them.

One of the first studies to successfully use HTMD for ligand binding was Buch et al. (Buch et al 2011). Using just 50 μs of simulation time, it was possible to reproduce the crystal binding pose, kinetics, and affinity of the binding of benzamidine to trypsin. However, while it was a critical proof-of-concept work, there were several aspects of the methods in that work which made it difficult to generalize to other systems. Others who worked on using MSMs for protein folding encountered similar successes and limitations, such as Bowman et al. found when studying Villin headpiece folding (Bowman et al 2009). While they could accurately approximate folding times, the found that RMSD based clustering was limited in part because structures that were close in RMSD may not interconvert rapidly, resulting in large heterogeneity inside clusters and hindering granularity of the MSM. Other clustering based on inter-atom contacts or distances, for example, has proved to be much more effective than spatial clustering.

Studies of folding and the motions of intrinsically disordered proteins provided additional difficulties. A critical problem in this case was that clusters can be far away geometrically (different conformations), but kinetically very close. This would result in too many conformations which are kinetically close for the MSM to identify. A projection method known as time-sensitive Independent Component Analysis (tICA) (Schwantes and Pande 2013; Pérez-Hernández et al 2013) was therefore incorporated into the process before clustering in order to alleviate this problem. tICA projects the data along its slowest varying coordinates, which can then be fed to the clustering algorithms. This almost universally improves the accuracy of the results.

With all these improvements, several studies have been able to show that HTMD can now be incorporated into a drug discovery pipeline. In an as yet unpublished work, we have recently measured affinity and kinetics for 42-fragment screen targeting Factor Xa, identifying crystal structures as well as secondary happy poses which are key to properly interpret experimental data on fragments. Expanding these methods to a full fragment library, typically on the order of 700 fragments, would be a simple question of cost and therefore time, considering the ever increase power of computing resources. Whether that is practical and cheaper than current best practices like X-ray and NMR spectroscopy remains to be answered.

Studies of binding in membrane proteins are also possible, even in difficult cases where the ligand is a lipid itself. In two studies by our group, we were able to show the binding pathway of lipid ligands binding to target proteins. In one work, we simulated the binding of the lipid anandamide to the enzyme FAAH (Dainese et al 2014). We also demonstrated how cholesterol interacts with and modulates the enzyme. In another, still unpublished work, we demonstrated the mechanism of binding of al lipid inhibitor, ML056, to sphingosine-1-phosphate receptor 1 (S1PR1). In that work, we were able to reproduce the crystallographic binding pose of the inhibitor, as well as characterize several important conformational changes along the pathway to binding. These studies were computationally intensive, requiring 250 μs and 800 μs of simulation respectively, and would be effectively impossible without a HTMD paradigm using standard hardware.

Drug design is more than just molecular recognition and binding. Often some fundamental activity of the protein remains to be understood before a drug discovery initiative can even start. MD has also been used to demonstrate that a postulated mechanism for HIV protease to cleave itself out of a long protein chain was indeed correct (Sadiq et al 2012). Novel behavior in the KID disordered protein (Stanley et al 2014) was also unveiled by a massive use of HTMD simulations. Accurately assessing the kinetic properties of both these systems was important and required 335 μs and 1.7 ms of simulation, respectively. And as a further example of how simulations can be important for therapeutic discovery, Shan et al. have used them to explain how mutations to EGFR lead to cancers (Shan et al 2012; Shan et al 2013).

## Conclusion

Computational tools are incorporated into the drug discovery pipeline because they speed up or corroborate experimental tests along the iterative cycle towards a drug. Molecular dynamics simulations are now accurate and fast enough that they can be used to help guide design choices of potential drugs. HTMD studies allow the full binding process of a compound to be sampled, giving important details on transient interactions, kinetics, affinity, and final resting pose. It can be used to test and rank an array of fragment compounds or analyze the binding of a lead compound. Further, they can be used to understand basic biophysical behavior that may be important before drug design even begins. As the raw power of individual GPUs increases and cloud services become ever cheaper and more routinely accessible, such studies will become even more commonplace.
